# Graphlet decomposition dataset of Tallinn’s road network from January 2020 OpenStreetMap data

**DOI:** 10.1016/j.dib.2025.111776

**Published:** 2025-06-13

**Authors:** Mahdi Rasoulinezhad, Nasim Eslamirad, Jenni Partanen

**Affiliations:** a Department of Civil Engineering & Architecture, Tallinn University of Technology, Tallinn, Estonia; b School of Architecture Planning and Environmental Policy, University College Dublin, Ireland; c Department of Built Environment, Aalto University, Otakaari 4, Espoo 02150, Finland

**Keywords:** Urban planning, Urban studies, Urban morphology, Graph theory, Network topology, Tallinn, Estonia

## Abstract

This paper presents a comprehensive dataset of graphlet decomposition for the road network of Tallinn, Estonia, based on OpenStreetMap (OSM) data representing the road network state as of 1 January 2020. Graphlets, which are small subgraphs, serve as powerful tools for analyzing and classifying local street structures in urban networks. The dataset includes counts of all possible four-node graphlet configurations for each intersection in the road network, provided in both Comma Separated Values (CSV) and Environmental Systems Research Institute (ESRI) Shapefile formats for maximum accessibility. The methodology for extracting these graphlets using Python and the Python ORbit Counting Algorithm (PyORCA) library is explained in details. The processing pipeline includes graph construction from spatial data, node-centric graphlet counting, and conversion back to geographic format. The resulting dataset enables researchers to identify recurring patterns in urban street networks, study urban morphology, and compare structural similarities between different urban areas. The code is designed for reproducibility, allowing researchers to apply the same analysis to other cities. This dataset contributes to the growing field of quantitative urban morphology and can support studies in urban planning, transportation network analysis, sustainable development, and comparative urban studies.

Specifications Table

**Table:** 

Subject	Earth & Environmental Sciences
Specific subject area	Graphlet Decomposition Dataset of Tallinn’s Road Network from January 2020 OSM Data
Type of data	Analyzed, Processed
Data collection	Road network data were collected from OSM via Geofabrik (https://download.geofabrik.de), using Shapefiles containing road features (files prefixed with gis_osm_roads_free). Historical datasets from Estonia for January 2020 were selected. Files were processed using custom Python scripts to extract graphlets from map data. Inclusion criteria focused on complete road segments; data extraction relied solely on open-source geospatial data and code.
Data source location	Institution: Tallinn University of Technology City/Town/Region: Tallinn Country: Estonia
Data accessibility	Repository name: Map Topology Direct URL to data: https://github.com/maraso-TTU/MapTopology/tree/main/output Data identification number: https://doi.org/10.5281/zenodo.15068582 Direct URL to images: https://github.com/maraso-TTU/MapTopology/tree/main/manuscript_images Direct URL to tables: https://github.com/maraso-TTU/MapTopology/tree/main/manuscript_tables
Related research article	No research article is related to the data preparation.

## 1 Value of the Data

• This dataset is based on the road network of Tallinn, Estonia, extracted from OSM data on January 1, 2020, providing a localized and time-specific representation of the urban network. Graphlet decomposition was applied to the 2020 road network of Tallinn, Estonia, using OSM data. This allowed for the identification of recurring local street structures, such as loops, stars, and chains, which reveal Tallinn’s urban morphology at a neighbourhood scale. Analysis of graphlet degree distributions allows for the comparison of Tallinn’s street patterns with other cities and the assessment of its structural uniqueness. These insights support urban planning by highlighting design patterns linked to historical growth and mobility potential, enabling data-driven comparisons across cities and time.
• This data supports the study of Sustainable Development Goals (SDGs) [1], specifically SDG 9: Industry, Innovation, and Infrastructure, and SDG 11: Sustainable Cities and Communities. It provides a reproducible method for analyzing and comparing urban street networks, aiding sustainable mobility planning. The graphlet configurations from Tallinn’s road network in early 2020 helps identify patterns that promote walkability and efficient transport, offering valuable insights for data-driven, resilient urban development.
• The dataset can be reused in GIS tools for spatial analysis or in network science software for structural comparison. A CSV version is also provided for statistical or machine learning modeling. Researchers can use this data to study neighbourhood-level morphology or track urban evolution by applying the same method to other years or regions using the provided code. Since the code is developed with reproducibility in mind for other researchers, it is possible to use the same code for other cities and compare Tallinn’s network structure with other cities.
• Generally, it is possible to input any Shapefile into the map folder of the provided code and obtain the graphlet decomposition in the output folder. Three main steps are needed to run the code. The first step is to install the requirements. The second step is to run the notebook. The third step is to set the bounding box of the geographical region within which graphlets should be extracted. A visualization is available in the notebook, allowing researchers to interactively choose the bounding box directly. In the code, graphlets are calculated for up to four nodes, but it is possible to compute them for up to five nodes.

## 2 Background

Infrastructure networks fundamentally shape urban form and function. While cities have always been structured by networks, their critical role in urban agglomeration and societal development has gained prominence only in recent decades [2,3]. Traditional network analysis methods like centrality measures, though widely used in urban studies, fail to capture localized, recurring structural patterns in street networks [[4], [5], [6]].

Graphlet-based methods address this limitation by focusing on small, connected subgraphs that identify and classify local street configurations. This approach provides a richer structural representation that reveals repeating motifs in urban networks—patterns that often correlate with social behavior, land use, and historical development [7,8]. By analyzing graphlet degree distributions, researchers can compare different cities' street networks through measuring local structural similarities.

This dataset applies graphlet decomposition to the January 2020 road network of Tallinn, Estonia, to capture localized structural patterns using OSM data. The resulting dataset serves as the foundation for a machine learning model. The scalability of this methodology makes it applicable to cities worldwide [5], enabling researchers to benchmark urban designs and study the evolution of urban forms over time [8].

## 3 Data Description

The project repository is organized into several directories containing the input data, analysis code, and output results. The complete structure of the project is detailed below:

Root Directory

• README.md: Project documentation and overview
• extractTopology.ipynb: Jupyter notebook containing the main analysis code
• requirements.txt: List of Python dependencies required to run the analysis
• maps/estonia2020/: Input data directory
• output/: Output data directory
• output_visualization.png: Visual representation of the analysis results

The project follows a clear hierarchical structure separating input data (maps directory), The project follows a clear hierarchical structure, separating input data (maps/ directory), processing code (root directory), and analysis results (output/ directory). The analysis results are provided in both CSV and Shapefile formats for flexibility in subsequent use.

### 3.1 Input Data

The input data is stored in a standard ESRI Shapefile format, containing road network information for Estonia as of January 2020. The dataset includes the following components:

• gis_osm_roads_free_1.shp: The main shapefile containing the geometric data of road networks
• gis_osm_roads_free_1.dbf: The attribute database file storing feature attributes
• gis_osm_roads_free_1.prj: The projection file defining the coordinate system (EPSG:4326)
• gis_osm_roads_free_1.cpg: The codepage file specifying character encoding
• gis_osm_roads_free_1.shx: The shape index file enabling quick spatial searches

### 3.2 Output Data

The analysis generates graphlet decomposition results in two formats:

1. CSV Format

• graphlet.csv: Contains the calculated graphlet frequencies for each node in the road network. Table 1 provides a sample view of graphlet.csv.

**Table 1:** A sample of graphlet.csv file.

coordinates	graphlet_0	graphlet_1	graphlet_2	graphlet_3	graphlet_4	graphlet_5	graphlet_6	graphlet_7	graphlet_8	graphlet_9	graphlet_10	graphlet_11	graphlet_12	graphlet_13	graphlet_14
(24.66445 59.39727)	0	3	7	3	0	15	14	5	1	0	0	0	0	0	0
(24.66459 59.39740)	1	4	7	6	0	16	21	4	4	0	0	0	0	0	0
(24.66482 59.39762)	2	4	11	6	0	19	33	9	4	0	1	0	0	0	0
(24.66527 59.39805)	3	3	7	3	0	17	14	5	1	0	0	0	0	0	0
• Structure includes:

− Coordinate information for each node

− 15 columns representing different 4-node graphlet frequencies

## 4 Shapefile Format

The results are also provided as a geographic Shapefile set:

• graphlet.shp: Contains point geometry for each node
• graphlet.dbf: Stores the graphlet frequency values
• graphlet.prj: Defines the coordinate system (EPSG:4326)
• graphlet.cpg: Specifies character encoding
• graphlet.shx: Index file for spatial queries

**Fig. 1:**
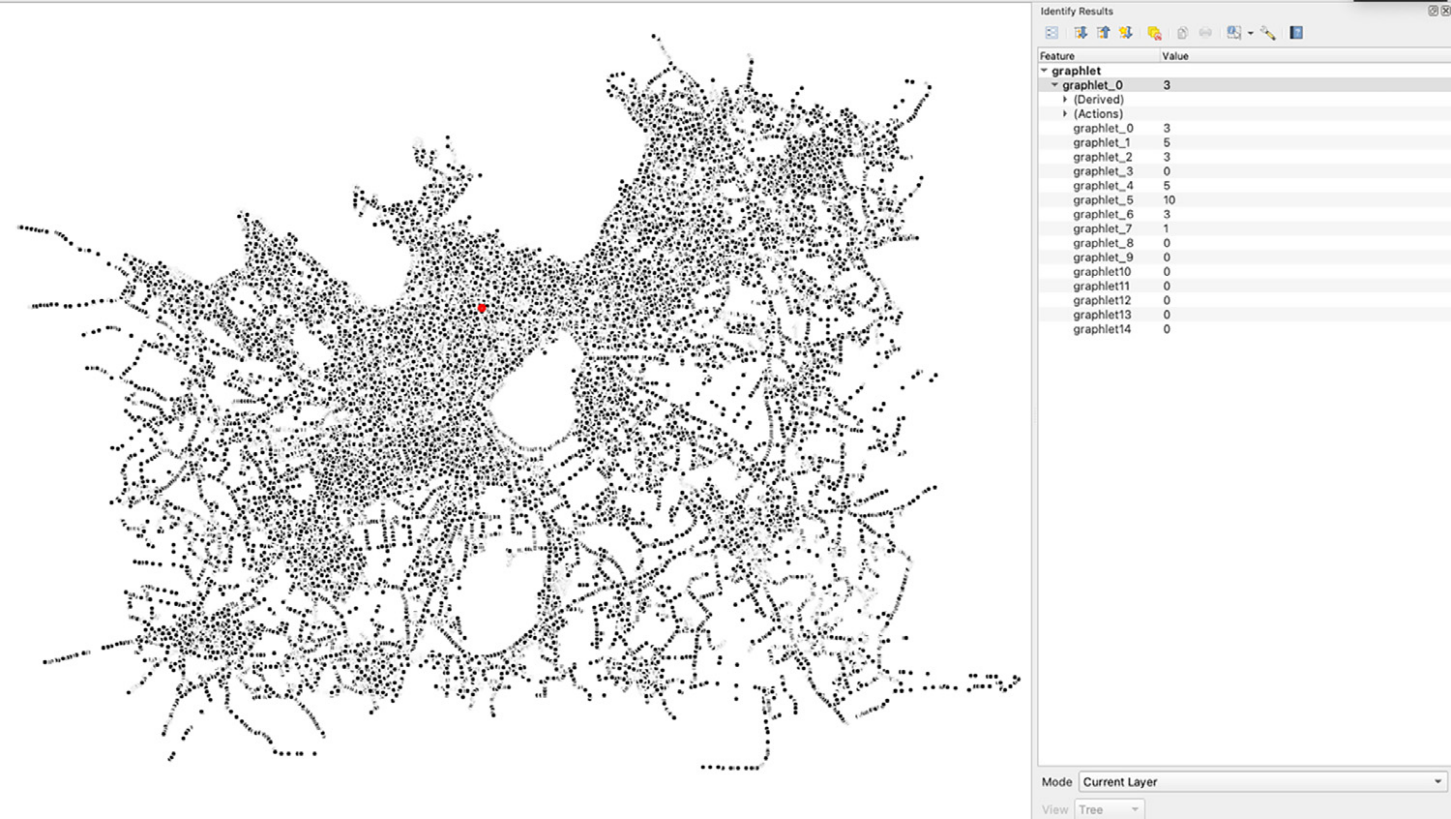
Graphlet decomposition of Tallinn’s 2020 road network, visualized from Shapefile format using QGIS.

Each node in the output files includes counts for all possible 4-node graphlet configurations found in the road network, represented as individual columns in both the CSV and Shapefile formats. The Shapefile is visualized in Figure 1.

## 5 Experimental Design, Materials and Methods

**Fig. 2:**
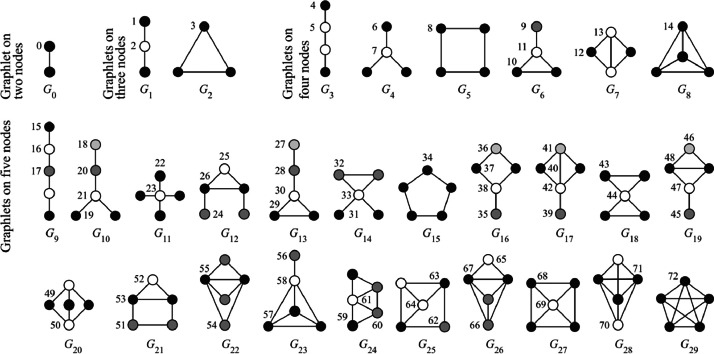
All graphlets that can be formed with up to five nodes. [Figure 2 is reproduced with permission from source: [9]].

The concept of graphlets was initially introduced by Pržulj [8] as a mathematical tool to compare different graphs based on the frequency of specific substructures, such as triangles, squares, or other small patterns. At the node level, graphlets describe the types of subgraphs connected to a node, making them uniquely suited for capturing local structural relationships within a network. Fig. 2 illustrates each unique small subgraph structure as a graphlet, with numbers assigned according to the naming convention established by Hočevar and Demšar [9].

To extract graphlets from maps, constructing a graph from the map data is the first step. Simply put, the next step is to count different subgraph structures connected to each individual node. This process, known as graphlet counting or graphlet decomposition, helps identify the distribution of subgraph shapes, sometimes referred to as graph motifs. Road network data was obtained from OSM for Estonia, focusing on the early 2020 dataset. The data was processed using Python 3.11 with the following key libraries:

• GeoPandas for spatial data handling
• NetworkX for graph operations
• Shapely for geometric operations
• PyORCA library for graphlet decomposition

Since historical OSM data is available only at the country level and consists of large files, a delineation step was incorporated into the code. The study area was defined using the following geographic coordinates to create a bounding box:

Western boundary: 24.5°E
Southern boundary: 59.3°N
Eastern boundary: 25.0°E
Northern boundary: 59.5°N

The code regarding this part is:

**Table:** 

A NetworkX graph was then constructed from the filtered road network data as follows:

• Nodes represent road intersections.
• Edges represent road segments.
• Original road attributes were preserved as edge attributes.

The graph construction process involved:

**Table:** 

Counting graphlets is a computationally intensive problem. As the graph size increases, the complexity grows, making it particularly challenging in urban networks with numerous nodes and edges. The Orbit Counting Algorithm (ORCA) is a combinatorial approach that enables efficient graphlet discovery and counting, even in large graphs [9]. Originally developed in C++, ORCA was later adapted into PyORCA to integrate graphlet extraction within the Python machine learning ecosystem [10].

Since NetworkX graphs cannot be used directly with PyORCA, two modifications are required:

• Nodes must be converted to integers for compatibility with the ORCA library.
• Edge lists must be generated in the pre-defined required format.

**Table:** 

The relationship between the number of nodes and possible graphlet shapes is defined in the size_table variable in the code. For example, as shown in Fig. 2, there are 15 distinct graphlet shapes possible with up to four nodes, and 73 graphlet shapes with up to 5 nodes. The graphlet_counts function is called with task='node' to count node-centric graphlets. For edge-centric graphlet counting, 'edge' can be specified as the task parameter. While it is technically possible to count graphlets up to size=5 and identify more complex subgraphs, these patterns are rarely observed in urban networks. Therefore, the analysis was limited to graphlets with up to four nodes. As a result, each node in graph G is assigned 15 additional attributes (graphlet_0 through graphlet_14), corresponding to the 15 different graphlet shapes, which are then integrated with the spatial data.

The graphlet counts were processed into a structured format:

• Results were converted to a pandas DataFrame
• Node coordinates were preserved
• Graphlet counts were labeled systematically (graphlet_0 through graphlet_14)

**Table:** 

The results were converted back to geographic format for visualization and further analysis:

• Point geometries were created from node coordinates
• A GeoDataFrame was constructed with the appropriate coordinate reference system (EPSG:4326), which is the default OSM coordinate system
• Results were exported to a shapefile

**Table:** 

The gdf looks like Table 2:

**Table 2:** A sample of the final dataframe.

graphlet_0	graphlet_1	graphlet_2	graphlet_3	graphlet_4	graphlet_5	graphlet_6	graphlet_7	graphlet_8	graphlet_9	graphlet_10	graphlet_11	graphlet_12	graphlet_13	graphlet_14	geometry
0	3	7	3	0	15	14	5	1	0	0	0	0	0	0	POINT (24.66445 59.39727)
1	4	7	6	0	16	21	4	4	0	0	0	0	0	0	POINT (24.66459 59.39740)
2	4	11	6	0	19	33	9	4	0	1	0	0	0	0	POINT (24.66482 59.39762)
3	3	7	3	0	17	14	5	1	0	0	0	0	0	0	POINT (24.66527 59.39805)

The final processed data was stored in the ESRI Shapefile format (.shp) with the following attributes:

• Geometric information (point features)
• 15 graphlet orbit counts per node
• Spatial reference system: EPSG:4326 (WGS 84)

**Fig. 3:**
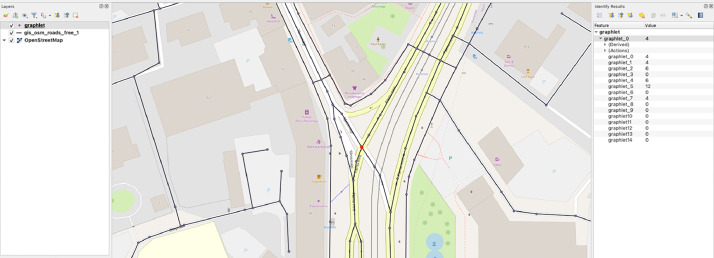
Graphlet decomposition of Tallinn’s 2020 road network overlaid on the road network, showing alignment with street structure. Each node contains attributes ranging from graphlet_0 to graphlet_14. Map made with QGIS.

The output Shapefile is easy to use as a layer in any GIS tool. It can be overlaid on the original input file, allowing researchers to utilize graphlet data directly or aggregate it within a specific region. Fig. 3 illustrates how the output Shapefile appears on top of the input layer.

The code is designed with reusability in mind. It can extract graphlet features from any Shapefile. Researchers can simply install the required dependencies, place the Shapefile in the designated map folder, and run the notebook. Defining the study area within the notebook improves processing efficiency, and the final Shapefile ensures seamless integration with GIS softwares.

## Limitations

The dataset is a static snapshot representing Tallinn’s street network in January 2020. It does not capture later developments or temporal changes. Despite the demonstrated utility of our graphlet decomposition approach for urban network analysis, several limitations warrant consideration. The quality and completeness of OSM data varies across regions, with inconsistent coverage, missing network elements, and variations in tagging practices potentially affecting extraction accuracy. Temporal limitations exist as our analysis represents a static snapshot of urban networks, failing to capture dynamic urban transformations. Additionally, validation remains challenging due to limited ground truth data, and the interpretability of graphlet-based findings for non-technical stakeholders presents ongoing difficulties. Finally, the Shapefile format of the output can be a limitation when using the output in some software; however, converting the final output to other geospatial formats like GPKG, GeoJSON, FlatGeobuf, and MapInfo with GeoPandas requires only a slight modification in the code.

## Ethics Statement

The authors have read and follow the ethical requirements for publication in Data in Brief and confirming that the current work does not involve human subjects, animal experiments, or any data collected from social media platforms.

## CRediT Author Statement

**Mahdi Rasoulinezhad:** Conceptualization, Methodology, Data Curation, Writing - Original Draft, Writing - Review &; Editing, Formal analysis. **Nasim Eslamirad**: Writin - Review & Editing, Formal analysis. **Jenni Partanen:** Supervision, Funding Acquisition, Writing - Review & Editing, Project Administration.

## Data Availability

GithubMapTopology (Original data).
